# **‘**Immunising’ physicians against availability bias in diagnostic reasoning: a randomised controlled experiment

**DOI:** 10.1136/bmjqs-2019-010079

**Published:** 2020-01-27

**Authors:** Sílvia Mamede, Marco Antonio de Carvalho-Filho, Rosa Malena Delbone de Faria, Daniel Franci, Maria do Patrocinio Tenorio Nunes, Ligia Maria Cayres Ribeiro, Julia Biegelmeyer, Laura Zwaan, Henk G Schmidt

**Affiliations:** 1 Institute of Medical Education Research Rotterdam, Erasmus MC, Rotterdam, Zuid-Holland, The Netherlands; 2 Psychology, Education and Child Studies, Erasmus University Rotterdam, Rotterdam, Zuid-Holland, The Netherlands; 3 Internal Medicine, State University of Campinas, Campinas, Brazil; 4 Center for Education Development and Research in the Health Professions, University of Groningen, Groningen, The Netherlands; 5 Propeudeutics, Federal University of Minas Gerais, Belo Horizonte, Brazil; 6 Education and Research Center, Santa Casa BH, Belo Horizonte, Minas Gerais, Brazil; 7 Internal Medicine, Universidade de São Paulo, Sao Paulo, Brazil; 8 Department of Medical Education Development, UNIFENAS Medical School, Belo Horizonte, Brazil

**Keywords:** cognitive biases, diagnostic errors, medical education, patient safety

## Abstract

**Background:**

Diagnostic errors have often been attributed to biases in physicians’ reasoning. Interventions to ‘immunise’ physicians against bias have focused on improving reasoning processes and have largely failed.

**Objective:**

To investigate the effect of increasing physicians’ relevant knowledge on their susceptibility to availability bias.

**Design, settings and participants:**

Three-phase multicentre randomised experiment with second-year internal medicine residents from eight teaching hospitals in Brazil.

**Interventions:**

Immunisation: Physicians diagnosed one of two sets of vignettes (either diseases associated with chronic diarrhoea or with jaundice) and compared/contrasted alternative diagnoses with feedback. Biasing phase (1 week later): Physicians were biased towards either inflammatory bowel disease or viral hepatitis. Diagnostic performance test: All physicians diagnosed three vignettes resembling inflammatory bowel disease, three resembling hepatitis (however, all with different diagnoses). Physicians who increased their knowledge of either chronic diarrhoea or jaundice 1 week earlier were expected to resist the bias attempt.

**Main outcome measurements:**

Diagnostic accuracy, measured by test score (range 0–1), computed for subjected-to-bias and not-subjected-to-bias vignettes diagnosed by immunised and not-immunised physicians.

**Results:**

Ninety-one residents participated in the experiment. Diagnostic accuracy differed on subjected-to-bias vignettes, with immunised physicians performing better than non-immunised physicians (0.40 vs 0.24; difference in accuracy 0.16 (95% CI 0.05 to 0.27); p=0.004), but not on not-subjected-to-bias vignettes (0.36 vs 0.41; difference −0.05 (95% CI −0.17 to 0.08); p=0.45). Bias only hampered non-immunised physicians, who performed worse on subjected-to-bias than not-subjected-to-bias vignettes (difference −0.17 (95% CI −0.28 to −0.05); p=0.005); immunised physicians’ accuracy did not differ (p=0.56).

**Conclusions:**

An intervention directed at increasing knowledge of clinical findings that discriminate between similar-looking diseases decreased physicians’ susceptibility to availability bias, reducing diagnostic errors, in a simulated setting. Future research needs to examine the degree to which the intervention benefits other disease clusters and performance in clinical practice.

**Trial registration number:**

68745917.1.1001.0068.

## Background

Diagnostic errors pose an important threat to patient safety.^1^ The diagnosis is estimated to be wrong 10%–15% of the time.^2^ While many errors have minor consequences, harm inflicted to patients is often serious,^3^ and diagnostic error remains the most common and most costly reason for malpractice claims in every large system.^2 4 5^ For example, a large study of claims in UK^6^ found failure or delay in diagnosis to account for 50% of the cases originated in primary care, with the death of the patient recorded in 21% of the cases.

Diagnostic errors are usually multifactorial, but errors in physicians’ *reasoning* have been detected in around 75% of the mistakes investigated in studies of malpractice claims^5^ and patients’ files.^7 8^ Such reasoning errors are frequently attributed to the use of heuristics, ‘rules of thumbs’ often employed by physicians, largely unconsciously, to make routine judgements.^9–11^ Usually efficient, heuristics may sometimes induce biases. For example, we often decide on the likelihood of an event (for instance a diagnosis) based on how easily examples of it come to mind.^12^ This usually helps but may induce *availability bias* when an *inappropriate* diagnosis comes more easily to mind. Availability bias caused errors when recent experiences with a particular disease^13 14^ made physicians confuse a subsequent case that looked like this disease (but had in fact another diagnosis) with the disease seen before. When irrelevant cues bring a wrong diagnosis to mind,^13–16^ if findings that are actually relevant remain unnoticed, an error will occur.^17 18^

There have been many interventions to ‘immunise’ physicians against bias. (We use the word ‘immunisation’ here as an apt metaphor for the characteristics of the intervention investigated in the study: (1) immunisation efficacy is always partial, which probably also applies to the intervention, and multiple doses are usually required to restore immunity; (2) immunisation is always disease specific, which also happens with a knowledge-based intervention; (3) immunisation increases resistance against a threat faced in future situations, an important point to highlight because our study is not concerned with interventions that support physicians at the moment of problem solving). These interventions have focused on improving the *process* of reasoning by increasing physicians’ ability to recognise circumstances that tend to induce bias and apply reasoning strategies to counteract bias. Courses on metacognitive skills, the basics of diagnostic reasoning and its possible cognitive pitfalls exemplify these interventions.^19–21^ Although such interventions eventually succeeded in increasing physicians’ awareness about biases,^19 20^ they have largely failed to change actual performance.^22 23^ Rates of diagnostic errors, whenever measured, remained unchanged.^24–27^

The present study deviated from these previous attempts by focusing on the *content* knowledge involved in diagnosis rather than the *process* of reasoning. We designed and tested an intervention directed at refining physicians’ knowledge of diseases, particularly knowledge of ‘discriminating features’. These features are findings that help distinguish between alternative diagnoses for a particular clinical presentation, because their presence substantially increases the likelihood of one of the diagnoses to be correct. Our assumption is that when this knowledge is robust, these features, when encountered in a case, will not be overlooked.^28^ This would tend to counteract the influence of irrelevant, bias-inducing cues. If this assumption is correct, immunising physicians against bias would require increasing the amount and organisation of physicians’ knowledge about these discriminating features.

To test this idea, we conducted an experiment in which an immunisation intervention was administered 1 week before a test that required physicians to diagnose clinical vignettes under conditions that were known to induce bias.^13^ We hypothesised that physicians who had gone through the immunisation phase would be less vulnerable to bias and demonstrate better diagnostic performance than ‘non-immunised’ physicians.

## Method

### Study design and setting

A multicentre randomised controlled experiment was conducted in eight teaching hospitals in five cities in Brazil from August 2017 to August 2018. Online supplementary 1 presents the study protocol.

10.1136/bmjqs-2019-010079.supp1 Supplementary data

**Figure 1:**
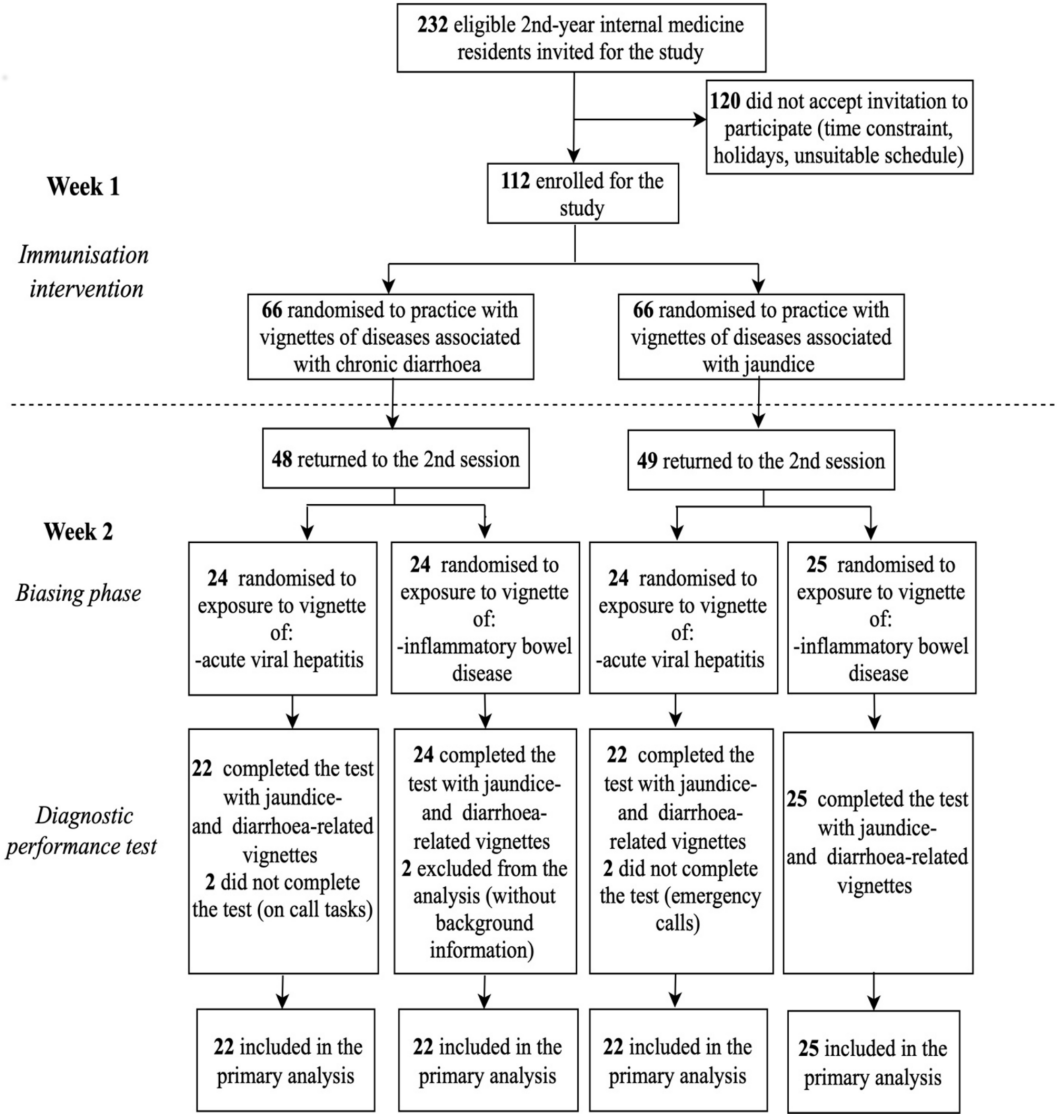
Diagram of the study and flow of participants.

The experiment consisted of three phases: an immunisation intervention, a biasing phase and a diagnostic performance test. In the immunisation phase, physicians diagnosed one of two sets of vignettes (either diseases associated with chronic diarrhoea or with jaundice) and compared and contrasted their diagnoses of these diseases, receiving feedback. The biasing and the test phases replicated a procedure that had been shown to induce availability bias in a previous study.^13^ In the biasing phase, physicians were exposed to a vignette of either inflammatory bowel disease (IBD) or acute viral hepatitis. Subsequently, in the test, all physicians diagnosed the same set of vignettes, half of them displaying diarrhoea-related diseases similar to IBD, the other half jaundice-related diseases similar to hepatitis, but all with different diagnoses. In the previous experiment, availability bias caused more mistakes to happen when the vignette was diagnosed after exposure to a similar-looking case in the biasing phase than when it was not (eg, physicians who encountered IBD in the biasing phase misdiagnosed the diarrhoea-related test vignettes as IBD more frequently than physicians who encountered hepatitis).^13^ In the present study, it was assumed that the intervention would ‘immunise’ physicians against bias either on the diarrhoea-related diseases or on the jaundice-related diseases (figure 1).

**Figure 2:**
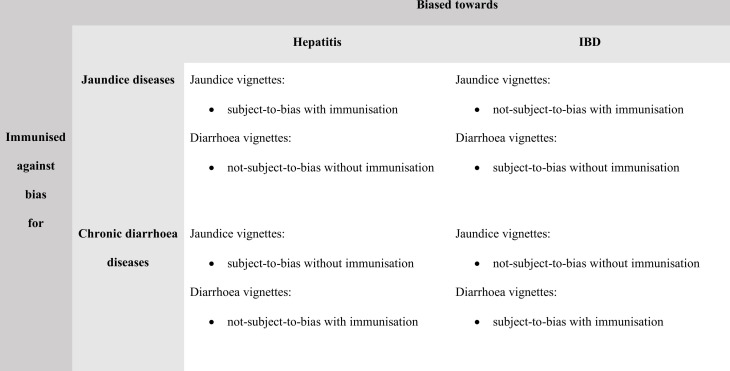
Types of test vignettes as a function of the diseases that the participant diagnosed in the immunisation intervention and the disease encountered in the biasing phase. IBD, inflammatory bowel disease.

The study involved therefore *two* different treatments. Each physician diagnosed the same vignettes in the test, but three test vignettes would look like the disease encountered in the biasing phase (hereafter ‘subjected-to-bias’ vignettes) and three would not (hereafter ‘not-subjected-to-bias’ vignettes) depending on the disease that the physician encountered in the biasing phase, and the physician would be either immunised or not immunised, depending on the diseases that the physician diagnosed in the immunisation intervention. (Notice that if bias depends on possessing specific knowledge, immunisation would also be specific to sets of related diseases.) For instance, for physicians who encountered hepatitis in the biasing phase, the jaundice test vignettes would be subjected to bias, while the diarrhoea test vignettes not subjected to bias. Among these physicians, those who diagnosed the jaundice vignettes in the immunisation intervention would be immunised against bias for the disease presented in the biasing phase, but not those who diagnosed the diarrhoea vignettes in the immunisation. The reverse would apply for the physicians who encountered IBD in the biasing phase. The combination of the two treatments would lead, therefore, to four ‘types’ of vignettes—subjected to bias with immunisation; subjected to bias without immunisation; not subjected to bias with immunisation; and not subjected to bias without immunisation—with each participant acting as each other’s control (figure 2).

### Participants

We recruited participants from the pool of internal medicine residents in the teaching hospitals. Residents in Brazil have an MD degree, obtained on completion of 6-year undergraduate education, after which they are allowed to engage in clinical practice. All residents enrolled in the second year of the training programme were considered eligible and invited by the programme director to voluntarily participate in the study (see online supplementary 1 section 2.2 for additional information). Written consent was obtained from participants.

### Sample size determination

A priori power analysis using to-be-detected effect of medium size (Cohen’s *f*=0.25) and the standard alpha level of 0.05 indicated that a sample size of 98 participants would be sufficient to achieve a power of 0.80.^29^ Enrolment rate was lower than expected, and data analysis was performed after completion of the planned sessions (see online supplementary 1 section 2.2 for additional information).

### Materials

The study used 25 written clinical vignettes prepared by board-certified internists (MACF, DF, MPTN, JB) based on real patients or by adjusting cases of previous studies.^13 14^ We aimed at using difficult cases to leave room for errors to occur. Two internists worked together to prepare each vignette, which was subsequently validated by the other internists. All vignettes contained sufficient information to arrive at the most likely diagnosis. Nine vignettes were ‘fillers’, used only to disguise the combination of diseases. Sixteen vignettes were relevant and actually considered for the analysis (we refer to the relevant vignettes hereafter). Half of the vignettes displayed diseases associated with jaundice and the other half diseases associated with chronic diarrhoea (online supplementary appendix 1). These diseases were chosen because, besides clinically important, they allowed us to use mostly vignettes validated in previous studies. In all phases, the vignettes were presented in booklets, each one prepared in two versions to counterbalance the presentation sequence.

10.1136/bmjqs-2019-010079.supp2 Supplementary data

### Intervention

The immunisation intervention consisted of two exercises carried out sequentially, combining deliberate reflection on clinical cases^30^ and feedback. Exercise 1 required physicians to diagnose a set of clinical vignettes, one by one, by following a procedure intended to increase knowledge of the clinical features that distinguish between diseases that share a similar clinical presentation. First, physicians read the vignette and gave the most likely diagnosis. Turning the page, they compared/contrasted alternative diagnoses presented in a table. They were requested to (1) list findings that speak in favour of their initial diagnosis, findings that speak against it and findings expected to be present if the initial diagnosis were correct but were absent in the vignette; (2) do the same for each alternative diagnosis; (3) rate the likelihood of each diagnosis under consideration; (4) underline findings shared by more than one diagnosis and circle those associated with only one of the diagnoses; and (5) list ‘discriminating features’, findings that help decide between the alternative diagnoses, because their presence is strongly associated with only one of them (see online supplementary 2 for an example).

In exercise 2, physicians received the same booklet but with the tables filled in, through a consensus model, by four expert internists (MACF, DF, MPTN, JB). For each vignette, participants compared their responses with the experts’ tables, underlying which discriminating features they had overlooked in exercise 1.

Two different sets of vignettes were used in the immunisation phase, one containing diarrhoea-related diseases and the other jaundice-related diseases (figure 1). Participants were randomly allocated to work either with the diarrhoea vignettes or with the jaundice vignettes (see online supplementary 1 for additional information). The intervention lasted 2 hours, with physicians proceeding through it in their own pace.

### Biasing phase and diagnostic performance test

The biasing phase and the test were conducted in a single session, purportedly as two independent studies. In the biasing phase, the physicians received a set of clinical vignettes, each one with a diagnosis, and indicated (in percentage) the likelihood that the diagnosis was correct. Two different sets of vignettes were used, each set containing the same four fillers (intended to hide the purpose of the biasing event) and one bias-inducing vignette, either IBD or acute viral hepatitis. Participants were randomly allocated to receive either one or the other set.

Subsequently, in the test, all participants received the same new set of vignettes. They were requested to read the vignette and write down the most likely diagnosis. Three vignettes displayed diseases that resemble IBD; three others resembled acute viral hepatitis, all with different diagnoses however.

Finally, the physicians provided demographic information and indicated how frequently they saw patients with the diseases included in the study by using a 5-point Likert scale (1=none; 5=very frequently).

### Outcomes

The primary outcome was diagnostic accuracy, measured by the score obtained in the test. Using a procedure proved reliable in previous studies,^13 14 31^ two board-certified internists (MACF, DF) independently and blindly classified all diagnoses provided for each vignette as correct, partially correct or incorrect (scored, respectively, as 1, 0.5 or 0). The inter-rater agreement was high (ICC=0.98). Discordant classifications were solved by discussion.

Additionally, we measured the occurrence of availability bias by counting the number of times that the disease of the bias-inducing vignette was mentioned as the diagnosis of the similar-looking test vignettes (IBD on the diarrhoea-related vignettes; hepatitis on the jaundice-related vignettes). This measurement was necessary to check if errors were actually caused by availability bias, because even physicians who had not encountered the similar-looking vignette in the biasing phase could incorrectly give its diagnosis to a test vignette that shares similar findings (eg, the test vignette of coeliac disease could be misdiagnosed as IBD even by physicians who saw hepatitis in the biasing phase).

### Data analysis

For each participant, we separately summed the diagnostic scores obtained in the test on the three subjected-to-bias vignettes and on the three not-subjected-to-bias vignettes. Mean diagnostic accuracy scores (0–1) were computed for each type of vignette. Similarly, the mean frequency (range 0–3) with which the diagnosis of the bias-inducing vignette was mentioned on the similar-looking test vignettes was computed for subjected-to-bias and not-subjected-to-bias vignettes. A mixed analysis of variance with immunisation against bias for the disease of the biasing phase (immunised vs non-immunised) as between-subjects factor and exposure to bias (subjected to bias and not subjected to bias) as within-subjects factor was performed on the mean diagnostic accuracy scores. This analysis assessed whether diagnostic accuracy decreases as a result of exposure to a similar-looking disease but is counteracted by the immunisation. Post hoc independent t-tests compared diagnostic accuracy of immunised and non-immunised physicians on the two types of vignettes (subjected to bias and not subjected to bias). Paired t-tests compared performance on each type of vignette within the same group of physicians. To verify whether availability bias actually occurred and was counteracted by immunisation, similar analyses were performed on the frequency with which the diagnosis of the bias-inducing vignette was given to the similar-looking test vignettes. Mean ratings of experience (range 0–5) with the diseases of the study were compared by performing independent t-test. All analyses were performed in SPSS V.25. The level of significance was set at two-sided p<0.05.

## Results

Ninety-one residents participated in the study (online supplementary 3, table 1). They reported moderate clinical experience with the diseases of the study, and there were no significant differences in participants’ characteristics at baseline (table 1).

**Table 1:** Baseline characteristics of physicians immunised and non-immunised against bias for the disease encountered in the biasing phase

	Immunised	Non-immunised	Overall
Age (years)			
Mean (95% CI)	27.39 (26.52 to 28.26)	27.91 (27.18 to 28.64)	27.67 (27.11 to 28.22)
Sex			
Male	23 (52%)	21 (45%)	44 (48%)
Female	21 (48%)	26 (55%)	47 (52%)
Experience with the diseases of the study (range 0–5)			
Mean (95% CI)	2.77 (2.65 to 2.90)	2.66 (2.48 to 2.85)	2.72 (2.61 to 2.83)

**Figure 3:**
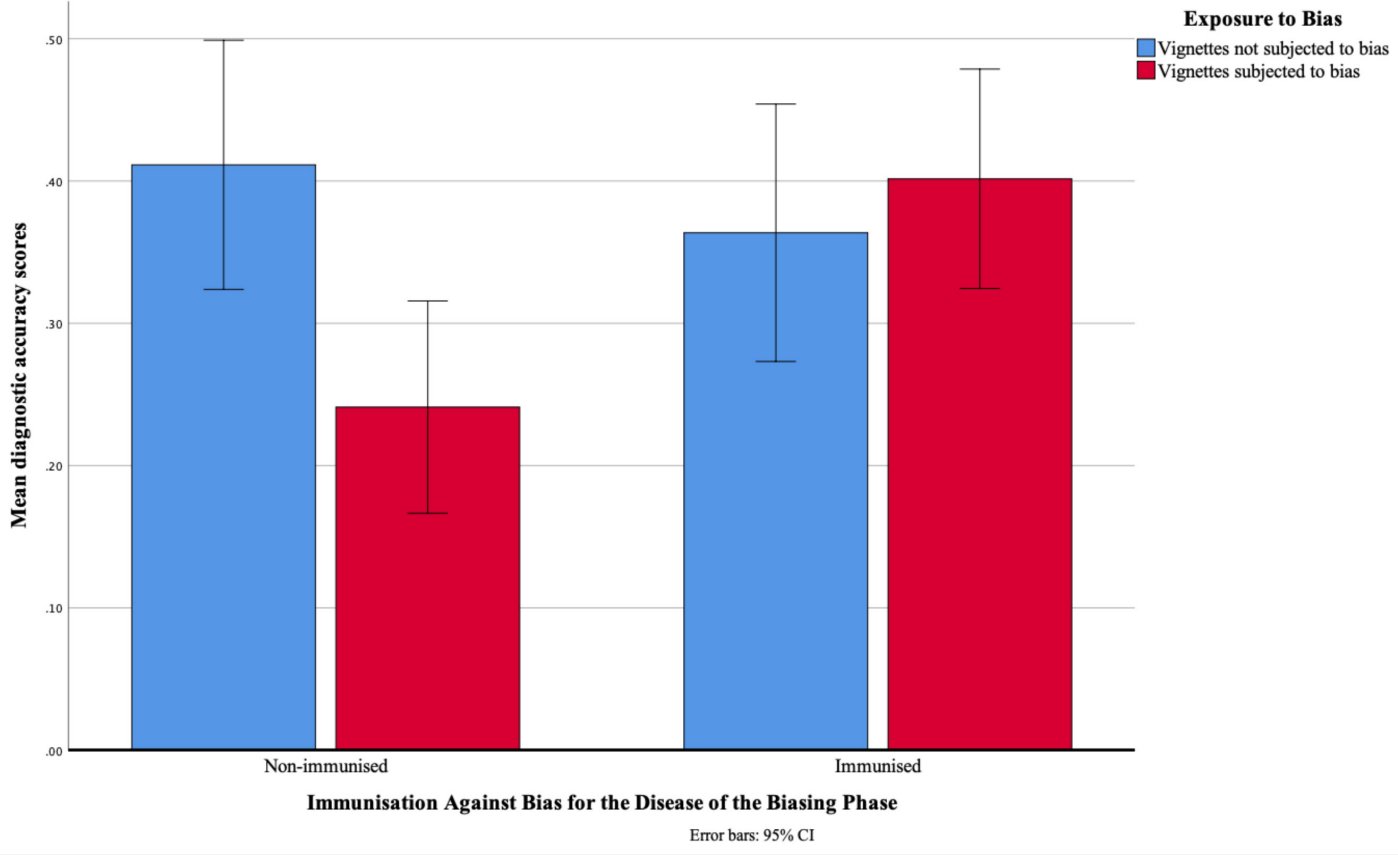
Diagnostic accuracy scores (range 0–1) as a function of previous exposure to a similar-looking disease in the biasing phase and immunisation against bias for the disease of the biasing phase.

Figure 3 presents the diagnostic accuracy scores obtained on subjected-to-bias and not-subjected-to-bias vignettes by immunised and non-immunised physicians. As expected, overall, diagnostic accuracy did not differ between not-subjected-to-bias and subjected-to-bias vignettes (respectively 0.39 vs 0.32; p=0.12; absolute difference in diagnostic accuracy 0.7 (95% CI −0.02 to 0.15)), nor between non-immunised and immunised physicians (0.33 vs 0.38; p=0.17; difference −0.06 (95% CI −0.14 to 0.02)), but there was a significant interaction effect (p=0.02). Post hoc analysis showed that the performance of immunised and non-immunised physicians only differed on subjected-to-bias vignettes. When diagnosis was preceded by exposure to a similar-looking disease (subjected-to-bias vignettes), physicians who had been immunised performed significantly better than those who had not (respectively 0.40 vs 0.24; p=0.004), with an absolute difference in diagnostic accuracy between the two groups of 0.16 (95% CI 0.05 to 0.27). On not-subjected-to-bias vignettes, immunised and non-immunised physicians did not significantly differ in accuracy (0.36 vs 0.41; p=0.45; difference −0.05 (95% CI −0.17 to 0.08)). Bias only hampered non-immunised physicians. They performed worse on subjected to bias than not subjected to bias on vignettes (p=0.005), with a difference in accuracy of −0.17 (95% CI −0.28 to −0.05), whereas the performance of immunised physicians did not significantly differ (p=0.56; difference 0.04 (95% CI −0.09 to 0.17)).

**Figure 4:**
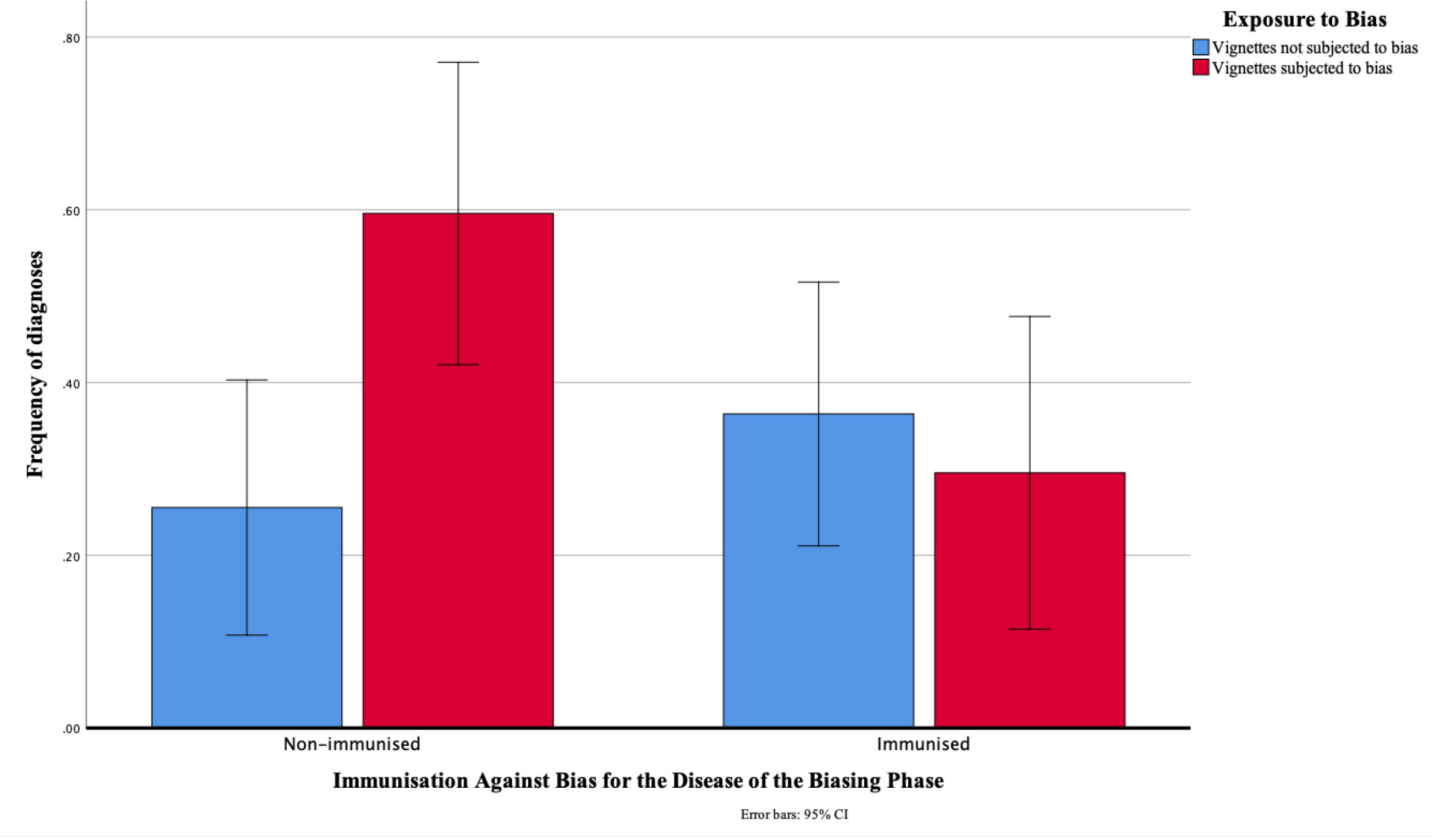
Frequency with which the diagnosis of the vignette of the biasing phase was incorrectly given to similar-looking test vignettes (range 0–3) as a function of exposure to a similar-looking disease in the biasing phase and immunisation against bias for the disease of the biasing phase.

Figure 4 presents the frequency with which the diagnosis of the bias-inducing vignette was given as the diagnosis of similar-looking test vignettes. Overall, the frequency did not differ between subjected-to-bias and not-subjected-to-bias vignettes (respectively 0.45 vs 0.31; p=0.13; difference in frequency 0.14 (95% CI −0.04 to 0.33)), nor between immunised and non-immunised physicians (0.32 vs 0.41; p=0.21; difference 0.10 (95% CI −0.05 to 0.24)). However, the interaction was significant (p=0.02). Test vignettes diagnosed after exposure to a similar-looking disease were more frequently confused with this disease by non-immunised than by immunised physicians (respectively 0.60 vs 0.30; p=0.02), with a difference in frequency of 0.30 (95% CI 0.04 to 0.56). When vignettes were not preceded by a similar-looking disease in the biasing phase (not subjected to bias), non-immunised and immunised physicians did not significantly differ in how frequently they mentioned the related diagnosis (0.25 vs 0.36; p=0.31; difference −0.11 (95% CI −0.32 to 0.40)). Only among the non-immunised physicians the frequency with which the bias-inducing diagnosis was mentioned for similar-looking test vignettes increased on subjected-to-bias relative to not-subjected-to-bias vignettes (p=0.01), with a difference of 0.34 (95% CI 0.08 to 0.60). Among immunised physicians, this frequency did not significantly differ (p=0.61; difference −0.07 (95% CI −0.33 to 0.20)).

## Discussion

An immunisation intervention directed at increasing physicians’ knowledge of a cluster of related diseases decreased the rates of diagnostic error when physicians diagnosed new vignettes of these diseases 1 week later under circumstances that are known to induce bias.^13 14^ After encountering one case of a disease, non-immunised physicians incorrectly gave that diagnosis to vignettes of different (though similar) diseases twice more frequently than immunised physicians. Consequently, diagnostic accuracy decreased 40% between immunised and non-immunised physicians. This difference in diagnostic accuracy was only observed on subjected-to-bias vignettes. Immunised and non-immunised physicians performed similarly on vignettes not preceded by exposure to a look-alike disease.

Taken together, these findings show that availability bias caused a substantial proportion of the diagnostic errors, and that the intervention counteracted the bias. The intervention required comparing and contrasting alternative diagnoses for look-alike diseases, focusing not on typical findings associated with a particular disease but on how that disease differs from other diseases that are frequent alternative explanations for a certain configuration of clinical findings. Psychological research^32^ supports the expectation that juxtaposing the alternative diagnoses and drawing attention to discriminating features would strengthen in physicians’ memory knowledge of critical features to be retrieved during differential diagnosis of these diseases. Robust knowledge of discriminating features would make a physician less likely to overlook them when irrelevant information, such as recent experiences with a similar-looking disease, brings an inappropriate diagnosis to mind. The findings suggest that this may have actually happened.

Although interventions exist that have been shown to reduce diagnostic errors^30 33 34^ or to counteract bias,^13 14^ all successful interventions up to now involve instructing physicians *while* they diagnose cases, such as priming them to review their initial diagnosis by engaging in deliberate reflection,^13 14^ using checklists^35^ or electronic support systems.^36^ Whereas empirical evidence exists of the effectiveness of these ‘workplace interventions’, interventions carried out *prior* to the diagnostic moment with the aim of increasing physicians’ resistance to bias in future situations have up to now shown no effect on rates of diagnostic errors.^23 27^ In the present study, the intervention made physicians less vulnerable to availability bias when they diagnosed, without receiving any particular instruction, new cases *1 week later*. Contrary to process-oriented ‘debiasing’ strategies,^21 22^ the intervention did not aim at recognition of bias-inducing cues but rather at recognition of critical diagnostic cues. Such intervention is therefore specific to sets of diseases that share a similar clinical presentation, consistently with the assumption that susceptibility to bias results primarily from lack of knowledge rather than from errors in reasoning. Note that the findings do not refute the potential influence of bias on reasoning, but they do show that specific disease knowledge counteracts such influence. Taken together with the hitherto limited effects of educational interventions aimed at improving reasoning processes on rates of diagnostic errors, our findings call for a new perspective in the search for strategies to increase physicians’ resistance to bias which gives attention to more knowledge-oriented interventions.^22^

The findings also reaffirm the potential of availability bias to cause diagnostic error. Exposure to only one case of a disease caused physicians to incorrectly provide this diagnosis to subsequent diseases that, though looking alike, were in fact different. The effect of the bias was not large but may increase when physicians encounter not one but several patients with similar presentations that are caused by different diseases, as it often happens in real settings such as primary care services or emergency rooms. Arguably, a wrong initial diagnosis generated under these circumstances may be repaired subsequently. However, the strongest predictor of final diagnostic accuracy is an accurate initial diagnosis,^37 38^ possibly because the initial hypothesis heavily influences subsequent information seeking. Physicians who generate an inaccurate hypothesis are more likely to fail to gather critical diagnostic information or to accurately interpret it, overvaluing neutral information as supporting the hypothesis while ignoring contradictory evidence.^39 40^ The studies showing lapses in physicians’ reasoning to be implicated in most diagnostic errors indeed suggest that an incorrect initial diagnosis is not easily overthrown.^5 7 41^

The intervention tested in the study has potential for adoption in practice in medical education. Many undergraduate and postgraduate programmes already have regular activities aimed at providing advanced students and residents with opportunity to practise with clinical problems. Exercises such as the intervention could be integrated into these activities, with trainees engaging in comparing and contrasting alternative diagnoses for similar-looking diseases. It would require selection of a set of frequent, relevant complaints and their usual clinical presentation, and organisation of practice around clusters of diseases that are usually alternative diagnoses for them. Organising such practice would require teachers to invest time and effort particularly for the development of appropriate cases, which may involve costs. On the other hand, the exercises themselves can be carried out independently by the trainees, without any particular supervision.

In the present study, one single exercise was enough to counteract the influence of availability bias, but further research is needed to determine the frequency with which trainees need to practise with the same cluster of diseases to ensure that the effect lasts. More research is required also to examine whether other target groups would also benefit from the intervention. Our participants were residents, and though it is likely that the intervention could be useful to advanced undergraduate students, this demands further investigation. Finally, the intervention showed to be effective to counteract availability bias. Other cognitive biases have been described,^10 11^ and though it is likely that they could also be counteracted by a knowledge-oriented intervention, this is still to be determined.

The study has limitations. First, the study was conducted in a simulated setting. The use of written vignettes, though shown by experimental research to be a good proxy for real settings performance,^42 43^ restricts generalisation of findings to real practice, where other cues would be available for the clinicians. On the other hand, while the vignettes contained all the information required for the diagnosis, in real practice physicians would need to search for the information themselves, and such search tends to be hindered by a wrong initial diagnosis.^39 40^ If bias caused error even when all the relevant information is given, the need to gather it would probably increase rather than reduce the damage. Second, our participants were residents with moderate experience with the diseases, and it is unclear if findings apply to experienced physicians. Whether experience per se makes physicians more or less susceptible to bias is unknown, as experienced physicians have more difficulties to revise initial hypotheses in light of disconfirming information,^44 45^ and escaping bias may depend not only on experience but also on specific features of disease knowledge. Experienced physicians would probably have more of this knowledge, and the intervention may turn to be less useful to them. Third, we tested the effect of the intervention after 1 week, the effect was considerable in light of what is at stake, but it may not last. Indeed, a single 2-hour exercise would probably not beat the influence of many other experiences that participants will go through in the course of their training. However, the study was a test in a simulated environment of an intervention that in real settings would involve not a single session but a longitudinal programme with regular similar exercises, which would tend to amplify learning. As it is the case for many vaccination schedules, multiple ‘doses’ of exercises such as the one tested in the study would probably be necessary for resistance to bias to be maintained across time. Finally, we studied availability bias, which was shown to occur and cause diagnostic errors in experiments^13 14 46^ and in retrospective reviews of errors,^47–49^ and it is unclear whether other cognitive biases could also be counteracted but a similar intervention.

In conclusion, an intervention directed to increase knowledge of clusters of diseases that are usually alternative diagnoses for a particular configuration of clinical findings, especially knowledge of findings that help discriminate between these diagnoses, made physicians less susceptible to availability bias when they diagnosed new cases after 1 week, reducing diagnostic errors. These findings suggest that the search for approaches to increase physicians’ resistance to bias, which are critical to minimise the burden of diagnostic error and improve patient safety, should focus on the development of knowledge-oriented interventions. Future research should investigate the effectiveness of the intervention in counteracting other types of cognitive biases and its value for experienced physicians.
