# Supplementary material for: ‘Immunising’ physicians against availability bias in diagnostic reasoning: a randomised controlled experiment
Source: BMJ Qual Saf. 2020 Jan 27;29(7):550–9. doi: 10.1136/bmjqs-2019-010079 (PMC7362774; doi:10.1136/bmjqs-2019-010079)
Supplement: Supplementary data [file bmjqs-2019-010079supp001.pdf]

ONLINE ONLY SUPPLEMENTARY FILES

Supplement 1 - Study protocol

Supplement 2 - Additional results

Supplement 3 – Example of a vignette used in the study (inflammatory bowel disease) in the immunisation intervention (Exercise 1)

Mamede S, *et al. BMJ Qual Saf* 2020; 29:550–559. doi: 10.1136/bmjqs-2019-010079

Supplement 1
Study protocol

Title: Strategies for the development of physicians' clinical reasoning and reduction of diagnostic error

Authors:

1. Sílvia Mamede, MD, PhD, Institute of Medical Education Research Rotterdam, Erasmus MC, University Medical Centre Rotterdam; Department of Psychology, Erasmus University Rotterdam, The Netherlands
2. Marco Antonio de Carvalho Filho, MD, PhD, Centre for Educational Research and Development in Health Professions, University Medical Centre, Groningen, The Netherlands; and Internal Medicine Department, School of Medical Sciences, State University of Campinas, Brazil
3. Rosa Malena Delbone de Faria, MD, PhD, Department of Propedeutics, Faculty of Medicine, Federal University of Minas Gerais, Belo Horizonte, Brazil
4. Daniel Franci, MD, Internal Medicine Department, School of Medical Sciences, State University of Campinas, Brazil.
5. Maria do Patrocinio Nunes, MD, PhD, Faculty of Medicine, University of São Paulo, Brazil
6. Ligia Maria Cayres Ribeiro, MD, MHPE, Department of Medical Education Development, José do Rosário Vellano University (UNIFENAS) Medical School, Belo Horizonte, Brazil.
7. Julia Biegelmeyer, MD, Faculty of Medicine, University of São Paulo, Brazil
8. Laura Zwaan, PhD, Institute of Medical Education Research Rotterdam, Erasmus MC, University Medical Centre Rotterdam
9. Henk G. Schmidt, PhD, Institute of Medical Education Research Rotterdam, Erasmus MC, University Medical Centre Rotterdam; Department of Psychology, Erasmus University Rotterdam, The Netherlands

1. Background

Diagnostic errors have attracted increasing attention since the 1999 Institute of Medicine report showed that between 44,000 and 98,000 people die annually in the United States due to avoidable medical errors.[1] If one takes the lower figure, deaths due to adverse effects caused by medical errors would supplant deaths due to traffic accidents, breast cancer or AIDS. A large fraction of these errors refers to treatment, but a substantial proportion consists of diagnostic errors, which involve a high cost, are potentially preventable, and have a high impact for both physicians and patients.[2] Diagnostic errors are found in all medical specialties at rates ranging from 5% in specialties of a more perceptive nature (e.g. radiology and pathology) to 15% in specialties such as emergency medicine and internal medicine.[3] Many of these errors can be corrected in time or produce minor adverse effects, but a substantial proportion leads to serious consequences, as autopsy studies have shown.[4] The Institute of Medicine report is usually seen as a milestone in the history of diagnostic error research, but the problem is not restricted to the United States, as subsequent studies in several countries have shown.[5] Although there are no large-scale studies of diagnostic error in Brazil, there is no reason to assume that the problem is less serious.

The literature distinguishes between three types of diagnostic error.[2] The “no-fault error” occurs in situations where the correct diagnosis could hardly be expected, for example, an extremely atypical presentation of a disease. The second type of error, known as a "system-related error", stems from failures in health services that affect physician performance, such as communication flaws. Finally, the "cognitive error" is one that can be attributed directly to the physician, resulting from a lack of appropriate knowledge, inadequate information collection or interpretation, inadequate verification or poor reasoning. Although multiple factors may interact to produce a diagnostic error, it has been repeatedly demonstrated that most of the errors are cognitive in nature. For example, a study of diagnostic errors in internal medicine conducted in US university hospitals showed flaws in the cognitive processes of physicians in 74% of cases.[6] Most of these errors were produced not because of lack of knowledge but because of deviations or flaws in clinical reasoning. Research on causes of diagnostic error in primary care services has reached the same conclusions, attributing the majority of errors to failures in the physician's reasoning.[7]

The reasons that make a physician run into flaws in clinical reasoning even though he would have enough knowledge to solve the problem have been the subject of much speculation. Such failures have often been attributed to cognitive biases associated with which has been named "non-analytical reasoning”.[8] As they gain experience, physicians tend to generate diagnostic hypotheses by rapidly recognizing similarities between the case in question and examples of previous patients (or prototypical scripts of diseases it has stored in memory), a process known as "pattern recognition".[9,10] What usually happens is that, in the first moments of a clinical encounter, characteristics of the patient "activate" in the doctor's memory scripts of one (or few) diseases, generating a diagnostic hypothesis. The elements of this illness script guide the physician in the subsequent process of seeking more information to verify whether the patient's findings are in fact compatible with the elements of the script.
This "pattern recognition" process occurs in a largely unconscious way, without involving effort, and is usually efficient. However, it seems to open the door to the occurrence of cognitive biases that can distort reasoning and lead to error.[11]

Many cognitive biases that may affect clinical reasoning have been described, but one of the most prevalent is the *availability bias*, which leads people to evaluate the likelihood of an event by the ease with which examples of this event come to mind.[12] Availability bias may produce diagnostic errors, for example, when exposure to media information or recent clinical experiences with a disease leads clinicians to diagnose similar (but in fact different) cases as the previously seen disease. By seeing, for example while on shift in an emergency room, a series of patients with influenza makes this diagnosis come to mind more easily when the physician encounters a close patient with similar symptoms, which can lead to error when the patient in fact has dengue fever. The literature on diagnostic error has suggested that availability bias is an important cause of cognitive diagnostic errors and at least two studies provide experimental evidence of this fact.[13,14] In one of these studies, internal medicine residents made more diagnostic errors in cases with similar clinical presentation (but different diagnosis) to cases they had encountered in a previous task.[13]

The recognition of the role of cognitive bias, such as the availability bias, in causing diagnostic errors has stimulated the search for interventions that make physicians less susceptible to such reasoning errors. One type of intervention that has been explored is to train physicians (or students) about possible biases, assuming that awareness of bias would reduce the diagnostic error.[15] Several formats of courses on clinical reasoning and cognitive

bias have been tried, for example, with residents of internal medicine or medical emergency, but the results have not been favourable. In the few studies in which diagnostic performance was assessed in subsequent tests, the intervention did not reduce the occurrence of diagnostic errors.[16] It has been questioned whether such intervention make sense, not only because of the results of these studies, which suggest a lack of effectiveness, but due to the very nature of cognitive bias. Because they derive from non-analytic reasoning, which takes place largely automatically, they are not subject to conscious control. Some authors have argued that cognitive biases must necessarily be related to the lack of sufficiently elaborate knowledge about the distinction between clinical presentations that look like but actually constitute different diseases.[16] Attention has therefore, it has been claimed, to be directed to the investigation of interventions focused on knowledge development, in particular the refinement of disease scripts that physicians have stored in memory and which are the basis of the diagnostic process.[15]

Better structured illness scripts, including knowledge of elements that allow differentiation between diagnoses that have similar clinical presentation, would make physicians less susceptible to bias and hence less prone to error. A deliberate reflection procedure on to-be-diagnosed cases, developed by Mamede et al.,[17,18] has been used to promote the refinement of illness scripts in a series of studies with medical students.[19,20] Briefly, the procedure involves comparing & contrasting different alternative diagnoses to the case in question, by means of a structured sequence of steps. In these studies, the students solved, during a learning session, the same set of cases, using the reflection procedure or making a differential diagnosis. The students who reflected on the cases in the learning session made fewer diagnostic errors when they resolved new cases a week later than the students who made the differential diagnosis. These findings suggest that, consistently with research in other fields,[21,22] the strategy of comparing & contrasting different scripts of alternative diagnoses for a to-be-solved problem leads to the refinement of illness scripts, making the clinician better able to distinguish between similar diseases in the future. If this effect also applies to more experienced trainees, such as residents, applying the deliberate reflection procedure during practice with clinical cases could contribute to prevent the occurrence of cognitive bias, such as availability bias, and reduce the occurrence of diagnostic error when physicians solve similar cases in the future.

Mamede S, *et al. BMJ Qual Saf* 2020; 29:550–559. doi: 10.1136/bmjqs-2019-010079

The present study aims to investigate the effectiveness of an immunization intervention based on deliberate reflection on clinical cases to reduce the negative effect of availability bias during the diagnosis of clinical problems.

Based on the aforementioned studies of availability bias and diagnostic error and on the influence of structured reflection on the learning of clinical diagnosis, it is expected that an intervention based on deliberate reflection acts as an "immunization" against the occurrence of bias, leading the following primary hypotheses:
(1) The prior exposure to cases of a given clinical presentation would induce availability bias during subsequent resolution of cases with similar clinical presentation but different diagnoses, leading to diagnostic errors and, consequently, to a lower diagnostic accuracy when these cases are resolved after exposure to a similar-looking disease ("subject-to-bias cases") than when they are resolved without prior exposure ("not-subject-to-bias cases").
(2) Previous practice with deliberate reflection on cases that share similar clinical presentation during an "immunization" intervention will reduce the deleterious effect of availability bias during subsequent resolution of similar-looking cases, leading to higher diagnostic accuracy in subject-to-bias cases that were seen during the immunization intervention than in subject-to-bias cases that were not seen during the intervention (i.e., "immunized physicians" would be less susceptible to availability bias and made fewer mistakes than "non-immunized physicians" when solving subject-to-bias cases, but, consistently with (1), no difference between immunized and non-immunized physicians would be observed in not-subject-to-bias cases).

2. Methods

2.1. Design

The present study is an experiment with two phases: an immunization intervention phase (session 1) and a test phase (session 2), one week after the first phase (see diagram of the study design in Appendix 1). The test phase consists of two tasks, a biasing phase and a diagnostic performance test. In the biasing phase, the physicians will first perform a "confirmation task," which requires evaluating the accuracy of the diagnosis given for a clinical case, having as chief complaint either chronic diarrhoea or jaundice. These cases are presented mixed with cases of non-relevant diseases, for which the same task is performed. All residents will subsequently diagnose eight new cases, four of which are similar to the

cases of the case of one of the syndromes seen in the biasing task and four similar to the cases of the second syndrome seen in the biasing task, but all with different diagnoses. This confirmation task (biasing task) has been shown in a previous study to induce availability bias and, consequently, diagnostic errors [13] However, in the present study, the residents will participate, one week before the experiment, in an immunization intervention consisting of practice, based on deliberate reflection, with clinical cases of one of the two clinical syndromes (either chronic diarrhoea or jaundice).

2.2. Participants

Participants in the study will be 98 second-year internal medicine residents from teaching hospitals in São Paulo and other cities (Appendix 2). Second-year residents are considered eligible for the present study because, as previous studies suggest, these professionals have, at this stage of their training, a similar expertise in a medical specialty that deals with a broad spectrum of problems and sufficient clinical experience to have developed pattern-recognition based on pattern recognition. The sample size was determined estimating a dropout rate of 20% between the two sessions. A prior power analysis, using to-be-detected effect of medium size Cohen's $f = 0.25$ (previous studies with similar interventions are not available), $\alpha = 0.05$, $\beta = 0.80$, for a mixed ANOVA with immunization as between-subjects factor (immunized or non-immunized) and biasing condition (subject-to-bias and not-subject-to-bias) as within-subjects factor.

All residents attending the second year of the internal medicine residency in each hospital will be invited by the program director to voluntarily participate in a study on interventions for improvement of diagnostic reasoning (see Appendix 2). Those who accept the invitation will be registered as participants. A code system based on self-determined codes will be used to ensure that responses are treated anonymously but allowing the connection of each participant's data in the two phases.

Potential adverse consequences to participants. Although there is no risk of participating in the study, the two phases will be carried out outside regular working hours, and it is possible that some of the participants feel fatigued by the additional work, although the activities have short duration. To avoid this problem, the activities will be carried out at the end of the week and the possibility to leave the activity at any moment will be assured to all the participants.

Benefits. Practice with a diversity of clinical cases is recognized as the primary mechanism in the development of clinical reasoning,[10] and participation in the study is expected to

Mamede S, *et al. BMJ Qual Saf* 2020; 29:550–559. doi: 10.1136/bmjqs-2019-010079

contribute to developing the diagnostic performance of residents. In addition, at the end of the first and second phase, participants will receive feedback that will demonstrate the rationale of an experienced internist to resolve each case. Each participant will have the opportunity to compare their own solution of the case with that of the expert, which should generate additional learning. Participants will also be informed about the theoretical basis of the study, during a lecture on the basics of clinical reasoning, cognitive bias and diagnostic error, performed immediately after the test phase. They will therefore have opportunity to gain additional knowledge about research findings in the area of medical expertise and clinical reasoning.

2.3. Materials and procedures

In total, 25 clinical cases will be used, 11 in the immunization phase and 14 in the test phase (5 for the confirmation task and 9 for the diagnostic task). Appendix 3 presents a breakdown of clinical cases according to each major complaint. Each case consists of a description (about 400 words) of a patient's medical history, history of the current problem, symptoms and findings of the physical examination, and diagnostic tests. The cases will be adapted from difficult cases used in previous studies,[13,17,18,23] all of them prepared by internists based on actual patients and with a confirmed diagnosis. In both phases of the study, the cases will be presented to participants in booklets, one by one.

In the immunization phase (session 1), the two versions of the booklets (either with diseases associated with chronic diarrhoea or with diseases associated with jaundice) will be randomly distributed to the registered participants (see Appendix 2); each participant will therefore practice with one of the two syndromes. The session is expected to last around 2 hours, and consists of two exercises. In the first exercise, for each case, participants are firstly asked to write the most likely diagnostic hypothesis for the case. The case is then re-presented on the subsequent page, and the participant is asked to reflect on the case by following a procedure that has been employed in previous studies, using a table provided to help register the results of reflection.[13,17] The participant is asked to 1) write the diagnosis previously given for the case; (2) read the case again and list the findings in the case description supporting this diagnosis, the findings that speak against it, and the findings that would have been expected to be present if that diagnosis were true but are not described in the case; (3) list alternative diagnoses that he/she would consider if the initial diagnosis generated for the case were found to be incorrect; (4) perform the same analysis (step 2) for each alternative diagnosis; (5) indicate his/her final decision about the most likely diagnosis; (6) and, finally, underline

in the table the findings that are shared by more than one of the diagnoses considered and circulate the findings that discriminate between these diagnoses. After completing the table, the participant is asked to list the findings that help discriminate between the alternative diagnoses because their presence (or absence) is strongly associated with only one of the diagnoses. After having solved all cases and completed the first exercise, participants move to the second exercise, which consists of comparing their reasoning on the case with that of an experienced internist. A new booklet presents each case again, one by one, together with the reflection table filled out by the internist, and the list of findings that discriminate between alternative diagnoses (tables will be prepared by the internists co-researchers, through a consensus model). The participant is asked to compare his / her diagnostic reasoning with the analysis of the case made by the internist.

In the test phase (session 2), lasting approximately 60 minutes, the participants will be randomly assigned to the biasing task either with a case of syndrome 1 or syndrome 2. Subsequently, all participants will diagnose the same 9 cases. The two tasks will be presented as two independent studies to minimize the chance that the confirmation task reveals the possibility of availability bias, consequently changing how residents would approach subsequent cases by inducing a more careful approach that does not reflect reasoning in practice situations. In the biasing task, 5 cases (1 bias-inducing case and 4 fillers) are presented one by one, with a diagnosis, and the participant is asked to indicate (in percentage) the probability that that diagnosis is correct. Subsequently, in what is presented to them as an independent study, all participants are asked to diagnose 9 cases, presented one by one. The participant should read the case and write the most likely diagnosis. Finally, participants are asked to provide background information (age, gender) and to indicate their clinical experience with the diseases used in the study by using a 5-point Likert-scale. Upon completion of the study, the participants receive feedback on the correct diagnosis of the cases.

2.4. Data analysis

The primary outcome of the study is the mean score of diagnostic accuracy in cases diagnosed under the following conditions: (1) "cases subject to bias and without previous immunization against bias for the disease of the biasing phase"; (2) "cases subject to bias with previous immunization against bias for the disease of the biasing phase " (3) "cases not subject to bias and with previous immunization against bias for the disease of the biasing phase "; (4) "cases not subject to bias and without previous immunization against bias for the

disease of the biasing phase". Besides diagnostic accuracy, for these types of cases, the frequency with which the diagnosis of the bias-inducing case in the biasing phase (i.e. the confirmation task) is mentioned on the similar-looking cases of the diagnostic task will be computed to evaluate the actual occurrence of availability bias. The comparison of the mean scores in each of the case types and the frequency of the diagnosis of the biasing task will allow us to evaluate, respectively, whether errors increase as a consequence of the bias-inducing task and are counteracted by the immunization, and whether these errors actually increased because availability bias occurred (and as counteracted by the immunization). This will examine whether the results observed in previous studies[13,14] are replicated and, in particular, if the immunization intervention was effective to prevent the occurrence of the bias and the resulting diagnostic errors.

For the computation of the diagnostic accuracy score, the accuracy of the diagnoses given by the participants will be evaluated considering the confirmed diagnosis of each case as a standard. All responses given by the participants to each case will be entered by a research assistant in a word file, without identification of the condition under which the response was provided thereby allowing for blind scoring. Two specialists in internal medicine will independently evaluate each diagnosis given by the participants, without knowing the condition under which they were given, as correct, partially correct or incorrect (assigning a score of 1, 0.5 or 0, respectively). A response will be considered correct whenever it mentions the core diagnosis of the case, and partially correct when the core diagnosis was not quoted, but a constituent element of the diagnosis was mentioned. This procedure has shown high levels of reliability in previous studies.[13,14,17] The interrater agreement will be assessed using a two-way mixed, absolute agreement, average-measures ICC. Differences in scores will be discussed by the two raters to reach a final score.

For each participant, mean diagnostic accuracy scores obtained on cases subject to bias and on cases not subject to bias will be computed. Similarly, the frequency with which the diagnosis of the bias-inducing case was given to similar-looking test cases will be computed on each type of case. Descriptive statistics will be computed for these two measures on subject-to-bias cases and not-subject-to-bias-cases. Two separate mixed ANOVAs with immunization against bias for the disease of the biasing phase (immunized or non-immunized) as between-subjects factor and exposure to bias diagnosing condition (subject to bias and not subject to bias) will be performed on these two outcome measures (i.e. diagnostic accuracy scores and frequency of the bias-inducing diagnosis). Significant effects

Mamede S, *et al. BMJ Qual Saf* 2020; 29:550–559. doi: 10.1136/bmjqs-2019-010079

will be further explored by performing independent and paired t-tests. Finally, descriptive statistics will be computed for participants' background characteristics and experience with the diseases used in the study and compared by performing Chi-square (for gender) and t-tests for age and experience.

Ethical approval

This study protocol will be submitted to the Research Ethics Committee of the University of São Paulo (CAPPESQ) and subsequently to "*Plataforma Brasil",* where the study is to be registered as a multicentred study.

Mamede S, *et al. BMJ Qual Saf* 2020; 29:550–559. doi: 10.1136/bmjqs-2019-010079

References

1. Kohn LT, Corrigan J, Donaldson MS. To err is human : building a safer health system. Washington, D.C.: National Academy Press; 2000. xxi, 287 p. p.

2. Graber M, Gordon R, Franklin N. Reducing diagnostic errors in medicine: what's the goal? *Acad Med* 2002;77(10):981-92.

3. Berner ES, Graber ML. Overconfidence as a cause of diagnostic error in medicine. *The Am J M* 200;121(5 Suppl):S2-23.

4. Graber ML. The incidence of diagnostic error in medicine. *BMJ Qual Saf* 2013 Oct;22 Suppl 2:ii21-ii7.

5. Weingart SN, Wilson RM, Gibberd RW, Harrison B. Epidemiology of medical error. *BMJ* 2000;320(7237):774-7.

6. Graber ML, Franklin N, Gordon R. Diagnostic error in internal medicine. *Arch Intern Med* 2005;165(13):1493-9.

7. Singh H, Thomas EJ, Khan MM, Petersen LA. Identifying diagnostic errors in primary care using an electronic screening algorithm. *Arch Intern Med* 2007;167(3):302-8.

8. Croskerry P. From mindless to mindful practice--cognitive bias and clinical decision making. *N Engl J Med* 2013;368(26):2445-8.

9. Schmidt HG, Norman GR, Boshuizen HPA. A Cognitive Perspective on Medical Expertise - Theory and Implications. *Acad Med* 1990;65(10):611-21.

10. Schmidt HG, Rikers RM. How expertise develops in medicine: knowledge encapsulation and illness script formation. *Med Educ* 2007;41(12):1133-9.

11. Redelmeier DA. Improving patient care. The cognitive psychology of missed diagnoses. *Ann Intern Med* 2005;142(2):115-20.

12. Tversky A, Kahneman D. Judgment under Uncertainty: Heuristics and Biases. *Science* 1974;185(4157):1124-31.

13. Mamede S, van Gog T, van den Berge K, Rikers RM, van Saase JL, van Guldener C, et al. Effect of availability bias and reflective reasoning on diagnostic accuracy among internal medicine residents. *JAMA* 2010;304(11):1198-203.

14. Schmidt HG, Mamede S, van den Berge K, van Gog T, van Saase JL, Rikers RM. Exposure to media information about a disease can cause doctors to misdiagnose similar-looking clinical cases. *Acad Med* 2014;89(2):285-91.

15. Schmidt HG, Mamede S. How to improve the teaching of clinical reasoning: a narrative review and a proposal. *Med Educ* 2015;49(10):961-73.

Mamede S, *et al. BMJ Qual Saf* 2020; 29:550–559. doi: 10.1136/bmjqs-2019-010079

16. Norman GR, Monteiro SD, Sherbino J, Ilgen JS, Schmidt HG, Mamede S. The Causes of Errors in Clinical Reasoning: Cognitive Biases, Knowledge Deficits, and Dual Process Thinking. *Acad Med* 2017 Jan;92(1):23-30.
17. Mamede S, Schmidt HG, Penaforte JC. Effects of reflective practice on the accuracy of medical diagnoses. *Med Educ* 2008;42(5):468-75.
18. Mamede S, Schmidt HG, Rikers RMJP, Custers EJFM, Splinter TAW, van Saase JLCM. Conscious thought beats deliberation without attention in diagnostic decision-making: at least when you are an expert. *Psychol Res* 2010;74(6):586-92.
19. Mamede S, van Gog T, Moura AS, de Faria RM, Peixoto JM, Rikers RM, et al. Reflection as a strategy to foster medical students' acquisition of diagnostic competence. *Med Educ* 2012;46(5):464-72.
20. Mamede S, van Gog T, Sampaio AM, de Faria RM, Maria JP, Schmidt HG. How can students' diagnostic competence benefit most from practice with clinical cases? The effects of structured reflection on future diagnosis of the same and novel diseases. *Acad Med* 2014;89(1):121-7.
21. Gentner D, Loewenstein J, Thompson L. Learning and transfer: A general role for analogical encoding. *J Educ Psychol* 2003;95(2):393-408.
22. Catrambone R, Holyoak KJ. Overcoming Contextual Limitations on Problem-Solving Transfer. *J Exp Psychol Learn* 1989;15(6):1147-56.
23. Mamede S, Schmidt HG, Rikers RMJP, Penaforte JC, Coelho-Filho JM. Breaking down automaticity: case ambiguity and the shift to reflective approaches in clinical reasoning. *Med Educ* 2007;41(12):1185-92.

Mamede S, *et al. BMJ Qual Saf* 2020; 29:550–559. doi: 10.1136/bmjqs-2019-010079

Appendix 1 - Diagram of the study design

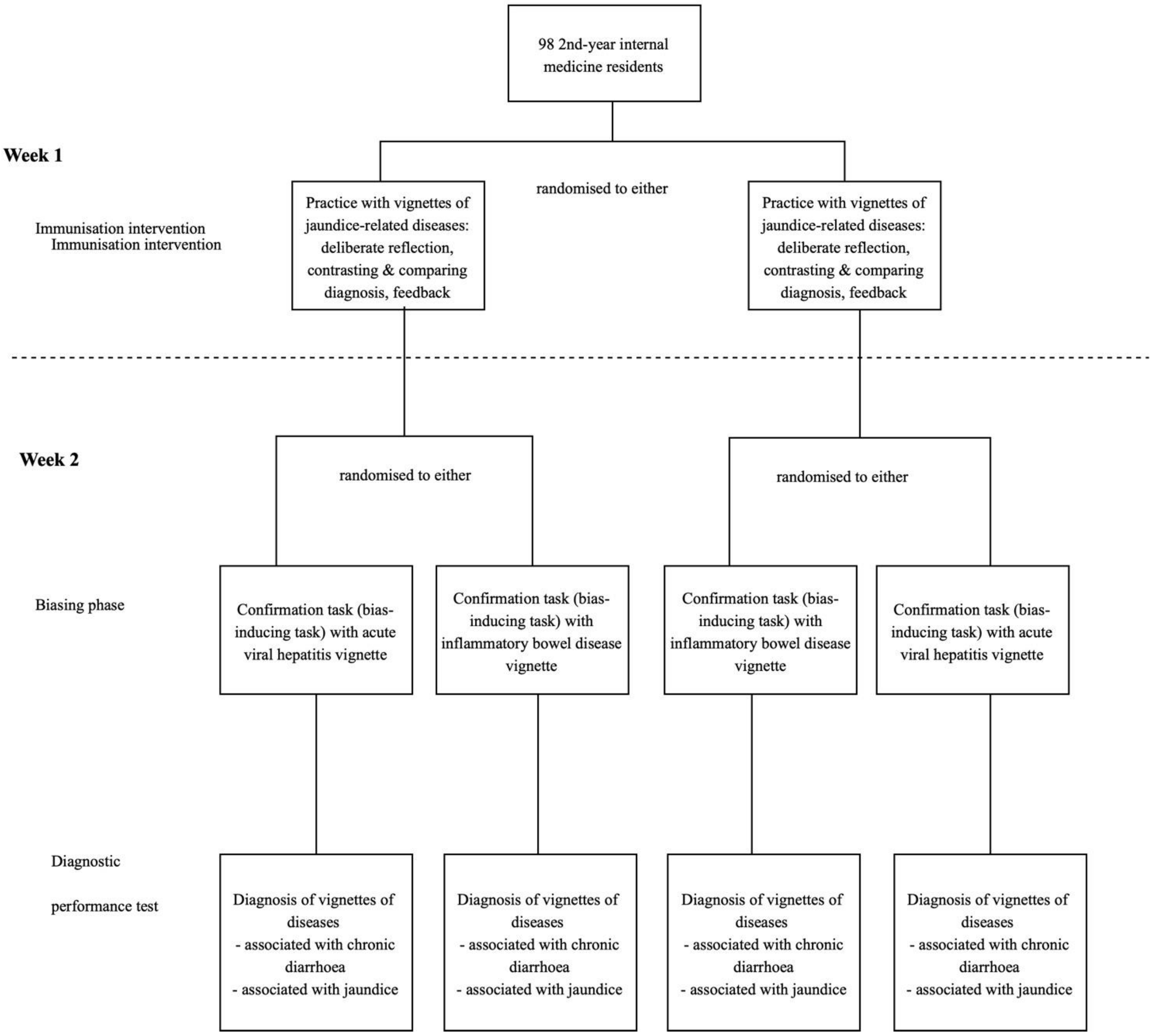

Mamede S, *et al. BMJ Qual Saf* 2020; 29:550–559. doi: 10.1136/bmjqs-2019-010079

Appendix 2 – Logistics and operational aspects

Cities and teaching hospitals involved in the study

City	Hospital	Eligible residents
Belo Horizonte	Santa Casa (41), Federal University of Minas Gerais (12), FHEMIG (40)	93
Campinas	UNICAMP	38
Fortaleza	HGF (13), Federal University of Ceará (16)	28
Manaus	Federal University of Amazonas	13
São Paulo	University of São Paulo	60
Total		232

An enrolment rate of 60 % is estimated, with variation expected due to local circumstances. A dropout rate of 20% between the first and the second session is expected.

Logistics

The study is estimated to be carried out in the course of one year, starting in the summer of 2017. Two sessions will be held in each hospital, by using local facilities regularly used for the training activities. The sessions will be booked by the director of the residency training considering the program schedule. The printing of booklets with be under the responsibility of the director of the residency training and will be carried out by using local regular printing schemas.

Procedure for randomization

Due to the difficulty to ensure that participants who accept to participate in the study actually attend the sessions and the need to maintain balance across the study, randomization will be ensure by having the four versions of the booklets prepared for each session randomly distributed to the attending residents. Prior to the session, the booklets to be used in the session will be put in individual envelopes and piled on blocks alternating the four versions of the booklets. After the residents are seated in the auditoriums, the envelopes will be handled to the attendees in the pre-arranged sequence, thereby ensuring that the distribution of participants within each condition remains balanced. The booklets will not contain any information that would identify the booklet as lined to a specific experimental condition.

Mamede S, *et al. BMJ Qual Saf* 2020; 29:550–559. doi: 10.1136/bmjqs-2019-010079

Appendix 3 – Diagnoses of the vignettes to be used in the three phases of the study

**Immunisation intervention**	**Biasing phase**	**Test phase**
Jaundice-related set		
Acute viral hepatitis	Acute viral hepatitis	Alcoholic cirrhosis
Alcoholic cirrhosis		Primary sclerosis cholangitis
Primary sclerosis cholangitis		Pancreas carcinoma
Pancreas carcinoma		
Chronic diarrhoea-related set		
Inflammatory bowel disease	Inflammatory bowel disease	Celiac disease
Celiac disease		Pseudomembranous colitis
Pseudomembranous colitis		Chronic infectious diarrhoea
Chronic infectious diarrhoea		
Fillers		
Rheumatoid arthritis	Stomach cancer	Nephrotic syndrome
Hyperthyroidism	Meningoencephalitis	Heart failure
Acute pyelonephritis	Chronic pulmonary obstructive disease	Acute appendicitis

Mamede S, *et al. BMJ Qual Saf* 2020; 29:550–559. doi: 10.1136/bmjqs-2019-010079

Supplement 2 – Additional results

Table 1 - Cities and teaching hospitals involved in the study

City	Hospital	Eligible residents	Enrolled	Completed
Belo Horizonte	Santa Casa, Federal University of Minas Gerais, FHEMIG	93	55	49
Campinas	UNICAMP	38	15	12
Fortaleza	HGF, Federal University of Ceará	28	18	10
Manaus	Federal University of Amazonas	13	11	10
São Paulo	University of São Paulo	60	13	10
Total		232	112	91

Table 2 – Initial and final diagnostic accuracy (range 0 – 1) in Exercise 1 of the immunization intervention on diseases associated with chronic diarrhoea and diseases associated with jaundice*

	**Initial diagnostic accuracy** Mean (SD)	**Final diagnostic accuracy** Mean (SD)	**Statistics**
Chronic diarrhoea	0·45 (0·30)	0·57 (0·30)	$t(43) = 3{\cdot}81$; $p < 0{\cdot}001$
Jaundice	0·46 (0·25)	0·61 (0·21)	$t(46) = 4{\cdot}73$; $p < 0{\cdot}001$

*Participants were randomly allocated to work either with vignettes with diseases associated with chronic diarrhoea or with vignettes with diseases associated with jaundice in the immunization intervention.

Supplement 3 – Example of a vignette used in the study (inflammatory bowel disease) in the immunisation intervention (Exercise 1)

Case 5

Read the following case and write down your initial diagnosis.

Male patient, 25-year-old, presents with complaints of diarrhoea over the last 4 weeks characterized by watery defecations around three times per day. He denies blood or mucus in the stools but complains of constant and uncomfortable abdominal pain in the lower abdomen on the left side. Since the beginning, he has fever which, despite having decreased intensity, is still present. He lost 8 kg in the period but is otherwise feeling well.

History: no significant pathologies were reported.

Physical examination: the patient is emaciated; weight 60 kg; height 1·65 m; BMI 20; Blood pressure 100 x 60 mmHg; heart rate = 90 bpm, regular pace. Head / neck: no abnormalities. Heart: regular heart rhythm with normal heart tones without heart murmurs. Lungs: normal and symmetric lung sounds; clear sounds on percussion. The abdomen is flaccid; the liver is palpable 1 cm below the right costal border with a smooth surface; the spleen is not palpable. The patient refers pain during palpation of the right side of the lower abdomen. There is no blood nor tumours on rectal examination. Extremities: necrotic, purulent lesion in the left ankle. The patient also complains of pain during the palpation of the left sacroiliac joint.

Laboratory tests: Haemoglobin = 78 g / L; White cells count: = 12.6 x 10^9/L; Eosinophils = 10%; Platelets = 160 x 10^9/L; ESR = 28 mm/hr; CRP = 5 mg/dL; TSH = 1.8 mU/L; AST = 30 U L; ALT = 25 U/L; Glucose (fasting) = 4.6 mmol/L; Faecal examination = no parasites.

What is the most likely diagnosis for this case?

Inflammatory Bowel Disease

Turn the page

Mamede S, *et al. BMJ Qual Saf* 2020; 29:550–559. doi: 10.1136/bmjqs-2019-010079

Case 5

The case is described here again

Male patient, 25-year-old, presents with complaints of diarrhoea over the last 4 weeks characterized by watery defecations around three times per day. He denies blood or mucus in the stools but complains of constant and uncomfortable abdominal pain in the lower abdomen on the left side. Since the beginning, he has fever which, despite having decreased intensity, is still present. He lost 8 kg in the period but is otherwise feeling well.

History: no significant pathologies were reported.

Physical examination: the patient is emaciated; weight 60 kg; height 1·65 m; BMI 20; Blood pressure 100 x 60 mmHg; heart rate = 90 bpm, regular pace. Head / neck: no abnormalities. Heart: regular heart rhythm with normal heart tones without heart murmurs. Lungs: normal and symmetric lung sounds; clear sounds on percussion. The abdomen is flaccid; the liver is palpable 1 cm below the right costal border with a smooth surface; the spleen is not palpable. The patient refers pain during palpation of the right side of the lower abdomen. There is no blood nor tumours on rectal examination. Extremities: necrotic, purulent lesion in the left ankle. The patient also complains of pain during the palpation of the left sacroiliac joint.

Laboratory tests: Haemoglobin = 78 g / L; White cells count: = 12.6 x 10^9/L; Eosinophils = 10%; Platelets = 160 x 10^9/L; ESR = 28 mm/hr; CRP = 5 mg/dL; TSH = 1.8 mU/L; AST = 30 U L; ALT = 25 U/L; Glucose (fasting) = 4.6 mmol/L; Faecal examination = no parasites.

A) The table below presents three possible diagnoses for this case in the column "Diagnostic Hypothesis". If the diagnosis you wrote on the previous page is not among these hypotheses, write it in the last line of the table in the "Diagnostic Hypothesis" column.
B) For each diagnostic hypothesis, write in the respective column the findings of the case that speak in favour of the hypothesis, the findings that speak against it and the findings that you would expect to find in the patient if this hypothesis were correct, but which are not present in the case.
C) After this analysis, evaluate the likelihood of each diagnosis under consideration. Write, in the "Likelihood" column, 1 for the most likely diagnosis for this case, 2 for the second most likely diagnosis, and so on.
D) Now, underline the findings that are present in more than one diagnostic hypothesis and then circle those that appear in only one of the hypotheses. Do this in the column "Findings that speak in favour of the diagnostic hypothesis".

Diagnostic Hypothesis	*Findings that speak in favour of the diagnosis*	*Findings that speak against the diagnosis*	*Findings expected to be found were the diagnosis true, but not present in the case*	*Likelihood*
Inflammatory Bowel Disease	Young patient, persistent diarrhoea, abdominal pain, fever, weight loss, anaemia, elevation of ESR and CRP, leucocytosis<br><br>Necrotic and purulent lesion in the left ankle (Pyoderma Gangrenous?)<br><br>Pain during the palpation of left sacroiliac joint	Absence of blood and mucus in the stool	Stools with blood and mucus<br>Inflammatory lesion present in imaging tests (colonoscopy, CT, MRI)<br>Compatible biopsy	1
Infectious Gastroenteritis	Persistent diarrhoea, fever, abdominal pain, increased ESR; increased CRP, leucocytosis	Eosinophilia, marked anaemia, absence of blood and mucus in the stool	Jaundice secondary to transinfectious hepatitis<br>Reactive arthritis<br>Positive stools culture	2
Celiac disease	Young patient, persistent diarrhoea, weight loss, abdominal pain, anaemia	Extra-intestinal lesions	Anti-Endomysial Antibodies and Anti-tissue Transglutaminase Antibody (anti-tTG)<br>Duodenal biopsy with villous atrophy	3

List the "discriminatory" findings, the most important ones to reach the diagnosis in this case: Persistent diarrhoea in a young man with positive inflammatory tests and abdominal pain, with extra intestinal lesions compatible with Pyoderma Ga
