# Supplementary material for: ‘Immunising’ physicians against availability bias in diagnostic reasoning: a randomised controlled experiment
Source: BMJ Qual Saf. 2020 Jan 27;29(7):550–9. doi: 10.1136/bmjqs-2019-010079 (PMC7362774; doi:10.1136/bmjqs-2019-010079)
Supplement: Supplementary data [file bmjqs-2019-010079supp002.pdf]

Appendix 1 -Diagnoses of the vignettes used in the three phases of the study

Inoculation intervention	Biasing phase	Test phase
Jaundice-related set		
Acute viral hepatitis	Acute viral hepatitis	Alcoholic cirrhosis
Alcoholic cirrhosis		Primary sclerosis cholangitis
Primary sclerosis cholangitis		Pancreas carcinoma
Pancreas carcinoma		
Chronic diarrhoea-related set		
Inflammatory bowel disease	Inflammatory bowel disease	Celiac disease
Celiac disease		Pseudomembranous colitis
Pseudomembranous colitis		Chronic infectious diarrhoea
Chronic infectious diarrhoea		
Fillers		
Rheumatoid arthritis	Stomach cancer	Nephrotic syndrome
Hyperthyroidism	Meningoencephalitis	Heart failure
Acute pyelonephritis	Chronic pulmonary obstructive disease	Acute appendicitis
